# Prevalence of Burnout in Intern Doctors on a Compulsory Rotational Internship in the Aftermath of the 2nd and 3rd Wave of COVID-19, Conducted in a Tertiary Hospital in Kolkata, India for the Academic Year 2021–2022

**DOI:** 10.1192/bjo.2022.355

**Published:** 2022-06-20

**Authors:** Sagarika Choudhury

**Affiliations:** R.G.Kar Medical College and Hospital, Kolkata, India

## Abstract

**Aims:**

Intern doctors are the backbone of the hospital infrastructure. While they are the first to provide patient care on an Emergency and Elective basis, they also happen to be the junior most. The interns of the year 2021–2022, apart from working in various departments of the hospital, were also the frontline workers in the 2nd and the 3rd wave of the COVID-19 pandemic. In the current global public health crisis, interns are more exposed to physical and mental exhaustion, owing to being overworked, along with carrying the burden of loss of patients, colleagues, and potentially infecting themselves and their loved ones to COVID-19. Burnout, a psychological syndrome that occurs due to work-related stress, includes emotional exhaustion (EE), depersonalisation (DP), and a sense of reduced personal accomplishment (PA). Intern doctors run a high risk of facing burnout—the prevalence of which is yet unknown. Hence, the survey was conducted.

**Methods:**

An online survey was carried out using MASLACH BURNOUT INVENTORY (MBI) among interns with their voluntary participation. 22 symptom items pertaining to occupational burnout were assessed, with three-component scales: emotional exhaustion (9 items), depersonalisation (5 items), and personal achievement (8 items), with a 7-level frequency scale for all MBI scales and 0–6 scoring.

Responses were received from 180 interns (n = 180). Questions regarding current department postings and contraction of COVID-19 were included in the survey.

**Results:**

Burnout was prevalent in most interns who tested positive for COVID-19 = 60% (108), followed by those whose family members tested positive for COVID-19 = 23.8% (43).

Burnout was seen more in female interns = 30% (54) than in males = 10.56% (19).

Burnout was seen more in interns working in the Emergency and Trauma = 41.67% (75), and the least in Ophthalmology = 1.67% (3).

**Conclusion:**

Burnout is significantly prevalent in intern doctors at the end of the academic year, especially due to the COVID-19 pandemic. Burnout can lead to increased medical errors, reduced patient satisfaction, which affects the quality of patient care. Understanding risk factors, improving workplace environment, limiting duty hours, workshops promoting healthy behaviours have been suggested to reduce burnouts and to prioritise both mental and physical health of interns so as to ultimately improve patient care.

